# Comorbidities in Anti-Cyclic Citrullinated Peptide Positive At-Risk Individuals Do Not Differ from Those Patients with Early Inflammatory Arthritis

**DOI:** 10.3389/fmed.2018.00035

**Published:** 2018-02-19

**Authors:** Sarah Twigg, Elena Nikiphorou, Jackie L. Nam, Laura Hunt, Kulveer Mankia, Peta Elizabeth Pentony, Jane E. Freeston, Ai Lyn Tan, Paul Emery

**Affiliations:** ^1^Bradford Teaching Hospitals NHS Foundation Trust, Bradford, United Kingdom; ^2^Leeds Institute for Rheumatic and Musculoskeletal Medicine, University of Leeds, Leeds, United Kingdom; ^3^Academic Rheumatology Department, King’s College London, London, United Kingdom; ^4^NIHR Leeds Biomedical Research Centre, Chapel Allerton Hospital, Leeds, United Kingdom

**Keywords:** comorbidities, inflammatory arthritis, rheumatoid arthritis, cyclic citrullinated peptide antibodies, at-risk of arthritis, depression and anxiety disorders

## Abstract

**Objectives:**

To compare comorbidities in a cohort of cyclic citrullinated peptide (CCP) antibody positive patients without or prior to onset of inflammatory arthritis (IA) to those in patients with early IA.

**Methods:**

Baseline data from two established cohorts were used. The first recruited people at risk of IA: CCP antibody positive cases without IA (CCP Cohort, *n* = 296). The second cohort [the Inflammatory Arthritis CONtinuum study (IACON)] recruited patients with early IA (*n* = 725). Proportions of patients with given comorbidities were compared between cohorts and then logistic regression was used to determine odds ratios (OR) for the CCP cohort having specific comorbidities, compared to IACON patients. Analyses adjusted for gender, age, smoking status, and body mass index.

**Results:**

Patients from the CCP cohort were younger (mean age 50, compared to 53 years). The proportion of patients with at least one comorbidity was higher in the IACON than the CCP cohort: (40% compared to 24%, respectively). Results of logistic regression analyses suggested the odds of hypertension, taking a lipid-lowering agent, ischemic heart disease, cerebrovascular disease, lung disease, and diabetes were not increased in either cohort. However, patients in the CCP cohort were more likely to be taking an antidepressant (OR = 1.62, 95% CI 1.03, 2.56, *p* = 0.037).

**Conclusion:**

There was no significant difference in comorbidities among people with CCP antibodies but without IA, compared to those of patients with established IA.

## Introduction

The presence of comorbidities in inflammatory arthritis (IA) has been linked with worse disease outcomes, including lower achievement of disease-remission (1) and premature death in the case of rheumatoid arthritis (RA), the commonest subset of IA (2, 3). The prevalence and impact of comorbidities on disease outcomes have been primarily studied in RA, with significant comorbidity having been reported at the outset of disease, increasing with follow-up (4).

UK data support a changing population demography over time with a rising prevalence of risk factors such as obesity (5), but also multimorbidity in general (6). Indeed, recent data have shown increasing age and level of comorbidity at disease presentation in two large RA inception cohorts spanning a 25-year period (7). The mechanisms underlying these secular trends are not easy to delineate and could be related to several factors, including changing population demographic trends, changing disease phenotype, and treatment use. It is acknowledged that earlier detection and screening for comorbidities may explain these trends.

Unlike extra-articular manifestations, which refer to non-articular features of IA, the term comorbidity in its strict sense is used to indicate the presence of an additional disease or condition, co-existing with the index disease (i.e., IA). Understanding the nature and timing/onset of comorbidities in patients with a predisposition to or with known IA can help provide insights into potential links to the index disease, e.g., common pathophysiological mechanisms and/or iatrogenic effects. This is becoming increasingly important in people with a predisposition to IA (which may be serological, genetic, or environmental) in order to identify and manage the comorbid and index disease with its associated pharmacotherapy. Unlike other forms of IA, some people at risk of seropositive RA can be identified by the presence of cyclic citrullinated peptide (CCP) antibodies, which may be detected up to 10 years prior to RA onset (8). Evaluating comorbidities in a cohort with a serological predisposition to RA assists in determining the onset of comorbidities prior to IA development. This formed the rationale for the study, which hypothesized that comorbidities start to accumulate in people at risk of IA. The study used two separate UK cohorts to investigate the timing and nature of comorbidities in people with a predisposition to IA, compared to established IA.

## Materials and Methods

### Patient Databases

This study is a comparison of two previously described inception cohorts: the CCP cohort study of patients with CCP antibodies, but without clinical IA, and the Inflammatory Arthritis CONtinuum, IACON, a study of patients with early IA. The CCP cohort study has been described previously (9). Briefly, patients in Yorkshire aged over 18 who presented to their primary care physician or other community service (e.g., physiotherapy) with a new musculoskeletal complaint were invited to participate. Patients were also recruited from the Leeds early arthritis clinic, if they did not have joint inflammation. Consented patients provided a blood sample for anti-CCP2 testing (using the commercially available immunocap method, Phadia, Sweden and Germany). Those with a positive result were invited to a research clinic based at Chapel Allerton Hospital in Leeds. Ethical approval was granted by the Leeds ethics committee (06/Q1205/169). Data collection began in 2007 and is ongoing, including follow-up of cases until the onset of IA. The present study used data recorded at the baseline visit from all cases included in the CCP cohort who did not have IA at the baseline visit. The IACON cohort has also been described previously (10). Patients aged over 18 who presented with early IA to Leeds Teaching Hospitals NHS Trust between 2010 and 2014 gave written consent to participate in IACON and attended data collection visits for 2 years. Ethical approval was granted by the Leeds (West) Research Ethics Committee (09/H1307/98). For the present analysis, baseline data from IACON cases with early IA and symptom duration of less than 24 months were included, except for 34 cases who had also participated in the CCP cohort study. We used data collected at the baseline visits of both studies. These data included current age and gender, whether the subject was a current, ex, or never-smoker, their body mass index (BMI), medical problems, and current medications.

### Comorbidity Data Collection

At the initial assessment, people in the CCP cohort were asked whether they had specific medical problems, namely hypertension, ischemic heart disease (IHD), cerebrovascular disease, chronic bronchitis or emphysema [recorded as chronic obstructive pulmonary disease (COPD)], asthma, diabetes (and how it was treated), renal or liver disease, inflammatory bowel disease, psoriasis, and gout. Participants were also asked whether there were any other conditions not mentioned in this list, and these were recorded as free text on the clinical record form. Current medications were also recorded. Data in this cohort were collected by rheumatologists or dedicated research nurses, who questioned participants and recorded their responses.

Baseline comorbidity data in IACON were collected by dedicated research nurses or clinicians at baseline. Patients were asked about specific diagnoses and their responses noted. In addition to the conditions asked about in the CCP clinic, patients were also asked whether they had any of the following: hypercholesterolemia, thyroid disease, depression, anxiety, cancer, peptic ulcer disease, epilepsy, demyelination, pseudogout, and osteoarthritis. As for the CCP cohort, names and doses of medications were also recorded. As there is a well-recognized association of IA with IHD, we also sought to identify differences in associations of the metabolic syndrome between the two cohorts (hypertension, dyslipidemia, diabetes, and IHD).

Data from both cohorts were validated by checking the lists of medications, which were recorded at all visits, against the comorbidities captured at baseline. Where the diagnosis for hypertension was missing but the patient was recorded as taking antihypertensive therapy for which no other reason was likely (e.g., a patient taking propranolol may have been using it for anxiety), the missing diagnosis was added accordingly. Participants in the CCP cohort were not asked about a history of hypercholesterolemia; therefore, this diagnosis was recorded only if treated, that is, if patients reported taking lipid-lowering therapy. Some people from both cohorts reported having both asthma and COPD, which likely reflects the nature of the respiratory disease; that is, those patients may have elements of both lung problems. Therefore, for the purpose of this analysis, asthma/COPD were collectively compared, that is, dealt with as a single comorbidity. Patients were not specifically asked about depression and anxiety during CCP cohort data collection but these problems were sometimes volunteered by the participants or it was noted that they were taking medication to treat these mental health conditions. In order to compare anxiety and depression across the two cohorts, we compared numbers of cases using antidepressant medication where antidepressants were prescribed in doses typically used to treat depression and not for pain modulation. As part of both studies, patients completed the EQ5D questionnaire (11), in which they were asked to indicate whether they considered themselves to be “not anxious or depressed” or “moderately anxious or depressed” or “extremely anxious or depressed.” We compared the proportions of cases who responded that they were moderately or extremely anxious or depressed at the baseline visit, across the two cohorts.

### Statistical Analysis

Descriptive statistics were used to compare baseline patient and disease characteristics between the two cohorts, using percentages for categorical variables and mean and SD for normally distributed variables. Individual comorbidities were compared across the two groups. To allow for confounders that may influence the presence of comorbidities in either group, a series of logistic regression models were constructed, with the presence of each comorbidity as outcome variables. In order to take into consideration some of the heterogeneity of the IACON cohort, which included patients with and without CCP antibodies, a sensitivity analysis was carried out in which comorbidities were compared between the CCP cohort and the 333 IACON cases who were CCP positive. Models were adjusted for: sex, age, cohort (that is, CCP cohort or IACON), whether the patient was a current or ex-smoker compared to those who had never smoked, and BMI. To allow for missing data, multiple imputation was used. Fifty datasets were imputed, using logistic regression for binary variables and predictive mean matching for BMI. The results were combined according to Rubin’s rules (12) and then compared to analyses of complete cases.

## Results

**Figure 1:**
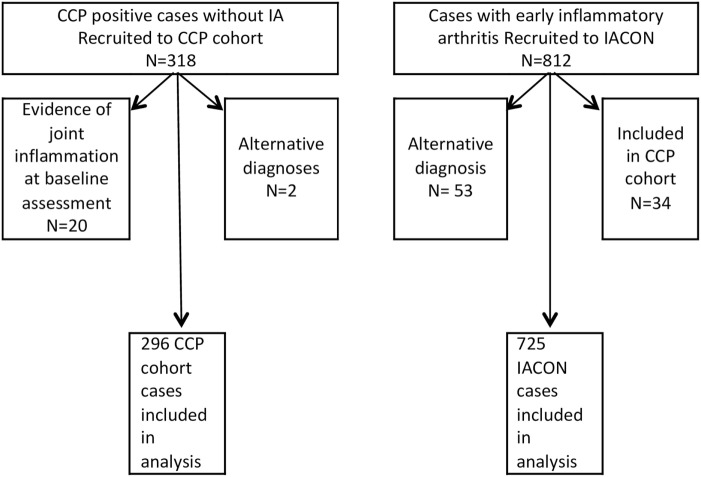
Diagram to show how numbers of cases were derived from each cohort.

Figure 1 shows how the numbers of cases included in the analysis were derived from those consented to the two cohorts. For 20 IACON cases and 2 cases from the CCP cohort, details on hypertension were amended from “missing” to “present” after the data were validated by comparing comorbidities to medications. In addition, in five cases from the CCP cohort, the diagnosis of hypertension was amended after it was judged to be inaccurate according to the drugs data and blood pressure recordings. In only five cases from the CCP cohort, the diagnosis of depression or anxiety was offered by the patient and recorded as an additional comorbidity; however, 33 participants were taking antidepressant medication for which no other indication (e.g., pain management) was likely from the diagnoses recorded in the database, and in these cases the diagnosis of depression was amended from “missing” to “present”.

Baseline characteristics of the two cohorts are given in Table 1, which also indicates how much of the data were missing. Participants in the CCP cohort were slightly younger: mean age was 49.8 years, compared to 52.8 years in IACON, and a greater proportion were female: 73%, compared to 68% in IACON. A greater proportion of the CCP cohort were current or ex-smokers and the mean BMI of this group was slightly higher (28.7, compared to 28.0). Of 271 cases from the CCP cohort, 78 (29%) progressed to IA, within a median time to progression of 34 months (IQR 15.5, 63.0). Within IACON, 468 (65%) of the cases met ACR/EULAR 2010 or ACR 1987 classification criteria for RA either at the baseline visit, or within 2 years of that visit.

**Table 1:** Baseline characteristics of the two cohorts.

	IACON (*n* = 725)				CCP cohort (*n* = 296)			
Baseline characteristics and comorbidities	Cases with characteristic		Cases with missing data for variable (%)		Cases with characteristic		Cases with missing data for variable (%)	
Female (%)	493	(68)	0 (0)		197	(73)	0 (0)	
Smoking: never	280	(39)	37 (5)		93	(34)	8 (3)	
Current	171	(24)			68	(25)		
Ex	237	(33)			102	(38)		
Mean age (SD)	52.8	(15.7)	0 (0)		49.8	(13.2)	0	
Mean BMI (SD)	28.0	(5.9)	26 (4)		28.7	(6.4)	73 (27)	
RF positive (%)	310	(43)	71 (10)		104	(38)	33 (12)	
CCP antibody positive (%)	333	(46)	10 (1)		271	(100)	0 (0)	
Cases with at least one comorbidity^a^ (%)	291	(40)	11 (2)		64	(24)	10 (4)	
Hypertension (%)	173	(24)	8	(1)	52	(19)	11	(4)
On lipid-lowering therapy (%)	128	(18)	21	(3)	38	(14)	60	(22)
Ischemic heart disease (IHD) (%)	32	(4)	8	(1)	10	(4)	12	(4)
Cerebrovascular disease (CVD) (%)	18	(3)	9	(1)	6	(2)	13	(4.8)
Peripheral vascular disease (%)	11	(2)	8	(1)	1	(0.4)		
COPD/asthma (%)	111	(15)	10	(1)	23	(8)	19	(7)
Diabetes mellitus (%)	48	(7)	5	(1)	12	(4)	15	(6)
Renal disease (%)	15	(2)	8	(1)	7	(2.5)	11	(4)
Chronic liver disease (%)	8	(1)	8	(1)	1	(0.4)	12	(4)
Associations of metabolic syndrome (%)^b^	239	(33)	24	(3)	71	(24)	80	(27)
Taking antidepressants (%)	67	(9)	21	(3)	33	(12)	60	(22)
EQ5D anxious or depressed (%)	173	(24)	8	(1)	52	(19)	11	(4)

*BMI, body mass index; CCP, cyclic citrullinated peptide; COPD, chronic obstructive pulmonary disease; RF, rheumatoid factor*.

*^a^Comorbidities were those specifically asked about in both cohorts: hypertension, IHD, CVD, chronic airways disease or asthma, diabetes mellitus, chronic renal disease, and chronic liver disease*.

*^b^Patients were considered to have associations of metabolic syndrome if they had hypertension, were taking lipid-lowering therapy, had IHD or diabetes*.

Table 1 also compares the percentage of patients with each comorbidity in the two cohorts. Most comorbidities were more prevalent in the IACON group, with a greater proportion of cases from IACON than the CCP cohort having each comorbidity, except for taking antidepressants, which was more common in the CCP cohort (12%, compared to 9% of patients in IACON). Of those taking antidepressants, one-third of those from the CCP cohort (11 of 33 people) responded that they were moderately or extremely anxious or depressed on the EQ5D questionnaire, and a similar proportion of cases from the CCP cohort not on antidepressants (29%) responded in the same way. A greater proportion of IACON cases taking antidepressants (66%) reported being moderately or extremely anxious or depressed, however, a similar proportion of IACON cases not on antidepressants (29%) responded in this way to the EQ5D questionnaire.

Table 2 gives the results of the logistic regression models. The odds of most comorbidities were not increased in either CCP or IACON cohorts, with the exception of antidepressants: reported antidepressant consumption was more likely in the CCP cohort, while reporting of moderate or extreme anxiety or depression was more likely in the IACON cohort. Table 2 also gives the results of the sensitivity analysis, in which comorbidities in the CCP cohort were compared to those in CCP antibody positive cases from the IACON cohort. This gave similar results to the analysis using the whole IACON cohort.

**Table 2:** Results of logistic regression models of the presence of comorbidities in the cyclic citrullinated peptide (CCP) cohort, compared to IACON.

Comparison of CCP cohort and all IACON cases					Comparison of CCP cohort and 333 anti-CCP positive IACON cases			
	
Comorbidity (outcome variables)	OR	95% CI		*P*	OR	95% CI		*P*
Hypertension	0.98	0.66	1.43	0.956	1.12	0.72	2.56	0.625
Lipid-lowering therapy	1.31	0.86	1.99	0.213	1.02	0.65	1.61	0.932
IHD	1.06	0.49	2.26	0.885	0.56	0.41	2.16	0.891
CVD	1.23	0.46	3.31	0.683	1.33	0.41	4.27	0.634
COPD/Asthma	0.76	0.50	1.17	0.216	0.67	0.42	1.08	0.100
Diabetes mellitus	0.75	0.38	1.49	0.414	0.81	0.38	1.72	0.591
Associations of metabolic syndrome^a^	1.12	0.78	1.62	0.539	1.26	0.84	1.88	0.262
Taking antidepressants	1.62	1.03	2.56	0.037	1.81	1.03	3.02	0.039
EQ5D anxious or depressed	0.57	0.41	0.78	<0.001	0.48	0.34	0.71	< 0.001

*CI, confidence interval; COPD, chronic obstructive pulmonary disease; CVD, cardiovascular disease; IHD, ischemic heart disease; OR, odds ratio; P (statistical probability)*.

*All models included the following covariates: gender, age, smoking status (ever smoked or currently a smoker compared to never smoked) and BMI*.

*^a^Patients were considered to have associations of metabolic syndrome if they had hypertension, or were taking lipid-lowering therapy, or had IHD or diabetes*.

## Discussion

To our knowledge, there are no previously published data on the prevalence of comorbidities in patients with a predisposition to RA. Through the CCP cohort, we have used routinely collected data from an at-risk population and compared this to a cohort of patients with early IA. Such studies are essential to understanding comorbidity burden, timing of comorbidities, and provide insights to underlying pathophysiological mechanisms in the development of comorbidities. We found that the prevalence of comorbidities in IACON were similar to those reported from a similar UK cohort of patients with early IA, the Norfolk Arthritis Register (13). Data from the Early Arthritis Study have indicated that comorbidities in RA increase with time, mainly in cardiovascular, non-cardiac vascular and respiratory systems, and furthermore, specific conditions (e.g., hypertension) occurred more frequently than in the general population (4).

The initial comparison of the two cohorts implied that specific comorbidities affected greater proportions of patients in IACON than the CCP cohort, but further analysis indicated that confounders, such as age, gender, smoking, and BMI, might explain this difference. Mean BMI was slightly higher in the CCP cohort, possibly because overweight and obesity are associated with common musculoskeletal conditions, such as osteoarthritis, for which patients are likely to see their primary care physician or a physiotherapist, and this was one route of recruitment for the CCP cohort. We found that the odds of taking an antidepressant was significantly greater for patients from the CCP cohort, compared to IACON (odds ratio, OR 1.62, 95% CI 1.03, 2.56), suggesting that more of these patients were diagnosed with and treated for anxiety and depression by their primary care physicians. Furthermore, the odds of a patient responding that they were extremely or moderately anxious or depressed at their baseline visit were lower in the CCP cohort (OR 0.57, 95% CI 0.41, 0.78, *p* < 0.001). The explanation for this paradoxical finding is not clear, but it is noteworthy that participants in either study were not asked about a prior history of anxiety and depression. IACON cases represented a group of patients with confirmed IA, many of whom had been treated with corticosteroids. So it is possible that with management of arthritis in IACON cases, symptoms of anxiety and depression improved and, thus, fewer patients from this cohort were taking antidepressants. Furthermore, corticosteroids may affect mood, with reports of both depression and euphoria, and these effects could not be taken into account in the present analyses. In comparison, cases from the CCP cohort were less likely to report feeling anxious or depressed at baseline EQ5D assessment, which is perhaps the effect of antidepressant treatment. There are important limitations here: first, the EQ5D data used only represented a single occasion for each patient, and furthermore, it is not suitable for the diagnosis of depression. Nevertheless, the finding was of interest and deserves further exploration. Within other cohorts, the psychological implications of an at-risk diagnosis have been reported. These have manifest as symptoms associated with anxiety and depression (14–16). Through focus group discussion and qualitative research techniques, researchers were able to demonstrate a theme of fear and uncertainty of a future diagnosis of RA (14, 15). Similar findings have been reported in first-degree relatives of RA (16). This may help to explain the increased prescription of antidepressants noted in our study.

### Limitations

This analysis compared data from two distinct cohorts, collected at the same center. There were differences in data collection between the two cohort studies, which may have biased the results. For example, there were different degrees of missingness in the two studies with more missing data in the CCP cohort than in IACON. To mitigate the impact of missing data, we used multiple imputation and 50 datasets to analyze our data. This will have helped to reduce bias due to missingness. The difference in missingness within the two studies is likely due to the way the comorbidity data were collected, which was mainly by dedicated research nurses in IACON and often by physicians in the CCP cohort. Our study was restricted to investigating specific comorbidities that participants were asked about and there were likely other comorbidities that were not captured. There could have been important differences in some comorbidities, such as cancer, but these were not included in the list of conditions to ask about in the CCP cohort. Some un-captured comorbidities, such as allergies, could have been indications for the use of corticosteroids and could, therefore, have been an additional source of unmeasured confounding. Where data were unavailable on hypercholesterolemia and depression/anxiety, we used pharmacotherapy as an indicator of disease. While this is not ideal, because it is possible that people may have these conditions without being on a drug treatment for them, this method of using pharmacotherapy as a proxy for the presence of a diagnosis is widely used (17).

Although the comorbidity data in this study were captured using a common questionnaire-style technique, this method is subject to recall bias. This is exemplified by the data validation exercise whereby we compared diagnoses to the recorded medications and found that patients were taking therapy for conditions that they had not disclosed. Hopefully, future comorbidity studies will make use of linkage to primary care databases, which contain contemporary and accurate details of consultations between patients and their general practitioners. The cohorts captured data on corticosteroid use at baseline and at follow-up assessments, but previous use of these medications was not asked about. Although this is a potential unmeasured confounder in our analysis, it is reasonable to assume that the IACON cohort were more likely to have received previous steroid for IA symptoms, and therefore the prevalence of diabetes could have been greater in IACON than the CCP cohort. As this association was not observed, previous use of steroid was unlikely to have had a significant impact on the outcome.

In conclusion, the comorbidities present in our CCP cohort were similar to those present in IACON, a cohort of patients with established IA. This indicates that comorbidities in IA may begin to accumulate before the onset of clinically apparent inflammation. This finding can be useful information to both patients and clinicians for whom the management of arthritis is more challenging in the presence of comorbidities. Our study also emphasizes the importance of early screening for comorbidities, including common mental health diseases such as anxiety and depression in patients presenting to the rheumatology clinic. Contemporary management of IA should incorporate a detailed assessment of comorbidities on disease presentation: this will inform treatment stratification and enable a more holistic approach to patient care.

## Ethics Statement

This study was carried out in accordance with the recommendations of the Leeds (West) Research Ethics Committee with written informed consent from all subjects. All subjects gave written informed consent in accordance with the Declaration of Helsinki. The protocol was approved by the “Leeds Research Ethics Committee.”

## Author Contributions

ST: design and conception of the research question for this paper, data analysis and writing of the manuscript, and final approval of the version to be published. EN: design and conception of the research question, critical review of analysis results, writing and review of the manuscript, and final approval of the version to be published. JN: conception and design of the CCP cohort, acquisition of data for the work, critical revision for intellectual content, and final approval of the version to be published. LH: design of the CCP cohort, acquisition of data for the work, critical revision for intellectual content, and final approval of the version to be published. KM: design of the CCP cohort, acquisition of data for the work, critical revision for intellectual content, final approval of the version to be published. PP: acquisition of data for the CCP cohort, critical revision for intellectual content, and final approval of the version to be published. JF: conception, design, and acquisition of data for the IACON cohort; critical revision for intellectual content; final approval of the version to be published. ALT: conception, design, and acquisition of data for the IACON cohort; critical revision for intellectual content; and final approval of the version to be published. PE: conception, design, and acquisition of data for the IACON and CCP cohorts, and critical revision for intellectual content.

## Conflict of Interest Statement

The CCP cohort study was also supported by AbbVie who provided funding for the anti-CCP testing.
